# Development of a Stand-Alone Independent Graphical User Interface for Neurological Disease Prediction with Automated Extraction and Segmentation of Gray and White Matter in Brain MRI Images

**DOI:** 10.1155/2019/9610212

**Published:** 2019-02-14

**Authors:** Ayush Goyal, Sunayana Tirumalasetty, Gahangir Hossain, Rajab Challoo, Manish Arya, Rajeev Agrawal, Deepak Agrawal

**Affiliations:** ^1^Texas A&M University-Kingsville, Kingsville, Texas, USA; ^2^G. L. Bajaj Institute of Technology and Management, Greater Noida, UP, India; ^3^All India Institute of Medical Sciences, New Delhi, India

## Abstract

This research presents an independent stand-alone graphical computational tool which functions as a neurological disease prediction framework for diagnosis of neurological disorders to assist neurologists or researchers in the field to perform automatic segmentation of gray and white matter regions in brain MRI images. The tool was built in collaboration with neurologists and neurosurgeons and many of the features are based on their feedback. This tool provides the user automatized functionality to perform automatic segmentation and extract the gray and white matter regions of patient brain image data using an algorithm called adapted fuzzy *c*-means (FCM) membership-based clustering with preprocessing using the elliptical Hough transform and postprocessing using connected region analysis. Dice coefficients for several patient brain MRI images were calculated to measure the similarity between the manual tracings by experts and automatic segmentations obtained in this research. The average Dice coefficients are 0.86 for gray matter, 0.88 for white matter, and 0.87 for total cortical matter. Dice coefficients of the proposed algorithm were also the highest when compared with previously published standard state-of-the-art brain MRI segmentation algorithms in terms of accuracy in segmenting the gray matter, white matter, and total cortical matter.

## 1. Introduction

Recent advances in neuropathology have significantly facilitated research into the underlying physiology in the advancement of cognitive impairment. This disorder is related to irregular protein buildup in the cerebrum, which prompts neuronal impairment in the synapses, nerve cells, and axons. Research has shown that anatomical changes start much before any symptomatic indications. The impairment starts in the medial temporal lobe, which contains the entorhinal cortex and hippocampus regions responsible for memory and motion and progresses to the neocortex region responsible for sensory perception, reasoning, and motor commands [1, 2]. The delayed symptoms of dementia are due to dissipation of cognitive reserve in terms of numbers of undamaged neurons, which result in loss of memory function only when decreasing below a certain limit.

### 1.1. Screening of Dementia

Memory loss related cognitive impairment precedes extensive damage in the temporal lobe, which over a limit is classified as Alzheimer's [3]. Early detection of predementia cognitive impairment through brain scans can facilitate therapy for slowing the progression of Alzheimer's or dementia starting early on. This is the motivation for early screening of cognitive impairment from brain images.

### 1.2. Brain MRI Cortical Measurements

Past research has demonstrated that assessment of cognitive impairment is feasible from measurement of the change or decrease in size of the cortical and hippocampal regions in anatomical MR images. Additionally, anatomical extraction, segmentation, and measurement of these regions in the medial temporal lobe from brain MR images can differentiate dementia from vascular or neurological degeneration. Furthermore, evaluation of the advancement of dementia via estimation of the rate of atrophy from the measurement of the abovementioned regions in the medial temporal lobe may be utilized to examine the efficacy of drugs administered to patients for the treatment of Alzheimer's from their MRI scans across several dates [4–6]. Structural anatomical changes in regions of the medial temporal lobe measured from brain MRI scans of patients taken over several dates can be used to estimate the rate of atrophy.

### 1.3. Measurement of Rate of Atrophy

Brain MR image-based measurement of the rate of atrophy in gray cortical matter is considered a legitimate marker of dementia or cognitive impairment. Atrophy of gray matter is an inescapable result of the degeneration of neuronal cells. The size or volume of cortical tissue is correlated to cognitive function, and the decrease in size or volume is proportional to the degree of cognitive deficiencies. Change in size of regions in the cortex maps to structural and anatomical change such as deposition of neurofibrillary tangle [7, 8] and neuropsychological deficiencies [9, 10]. The first noticeable changes in structural MRI occur along the polysynaptic connections between the hippocampus, entorhinal cortex, and posterior cingulate cortex, due to atrophy caused by excessive protein buildup. This atrophy of the synapses is expected and predictable, given the memory loss in patients in early stage cognitive impairment [11, 12]. At a more advanced stage, neuronal degeneration in temporal, frontal, and parietal lobes results in impairments in speech, motion, and behavior [13, 14]. Complete cerebral [15–19], entorhinal cortical [20], hippocampal [9,21–23], and temporal [24, 25] lobe volume atrophy rates and swelling of ventricular regions [9, 21, 23, 26] both correspond to cognitive impairment, validating their legitimacy as measures of dementia. The utilization of an image-based measure of cognitive degeneration requires that its progression is known at the diverse phases of the disorder and that its association with other imaging-based markers is accounted for. The rate of atrophy varies with the severity of the dementia, from mild cognitive impairment all the way to Alzheimer's disease. In the advanced stages of dementia, anatomical measures have higher sensitivity to atrophy than biomarkers of the proteins imaged or analyzed in cerebrospinal fluid samples [18, 27]. In the presymptom to mild impairment phases, amyloid protein biomarkers show more anomalies than anatomical or structural measures [16,28–33]. Anatomical degeneration occurs at both the macro (tissue level) and micro (axon, dendrites, myelin, and neuron level) scales. These atrophic changes are quantifiable through MR spectroscopy, magnetization transfer, fiber tracking, and diffusion weighted imaging [34–38]. Functional and tissue perfusion MR imaging also can be employed as screening measures [39–42] but require more extensive clinical validation as of present.

### 1.4. Brain Segmentation

The neocortex of the cerebrum in the MRI scan can be divided into regions after image registration. To perform this, a labeling procedure can be utilized to label each pixel in the cortex to be in one of the regions [43]. Also, clustering based pixel classification algorithms can be used to segment the amygdala and hippocampus regions, which are no neocortical. Algorithms used for the segmentation can either be region growing or pixel clustering using the similarities and disparities in pixel intensities and neighborhood connectivity [44].

### 1.5. Cortex Thickness Measurements

In patient brain MRI images, the thickness of the cerebral cortex is one of the important parameters used for assessing dementia. The cerebral cortex thickness is measured as the orthogonal distance across the edges between the gray matter and white matter and cerebrospinal fluid. The thickness is measured at each point axially across the full cortical mantle [45–47]. Validation of cortical thickness estimation method has been accepted by means of histological [48] and manual estimations [49]. Cortex thickness measurement is across gray and white matter edges and hence requires gray and white matter segmentation. This paper presents development of an algorithm for automatically calculating the gray and white matter region boundaries postsegmentation, after which computation of the thickness and volume of the cortex for assessing dementia or cognitive impairment in patients can be done. Future work would entail validating the accuracy of the cortex thickness measurements using distance across the boundaries and testing for robustness of the algorithm over varying image acquisition systems with changing scanner type, signal to noise ratio, and number of MRI slices captured [50, 51].

## 2. Background and Motivation

Nowadays, with the increase in patients with brain abnormalities, analyzing the patient's brain MRI images by extracting diagnostic features and other clinical information is the most challenging task for doctors or neurologists in the field of biomedical image processing. This work presents automatic segmentation of gray and white matter regions as anatomical features in brain MRI images. Changes in the size or volume of these regions can be correlated to changes in cerebral structure in patients with Alzheimer's, dementia, cognitive impairment, or other neurological disorders.

**Figure 1:**
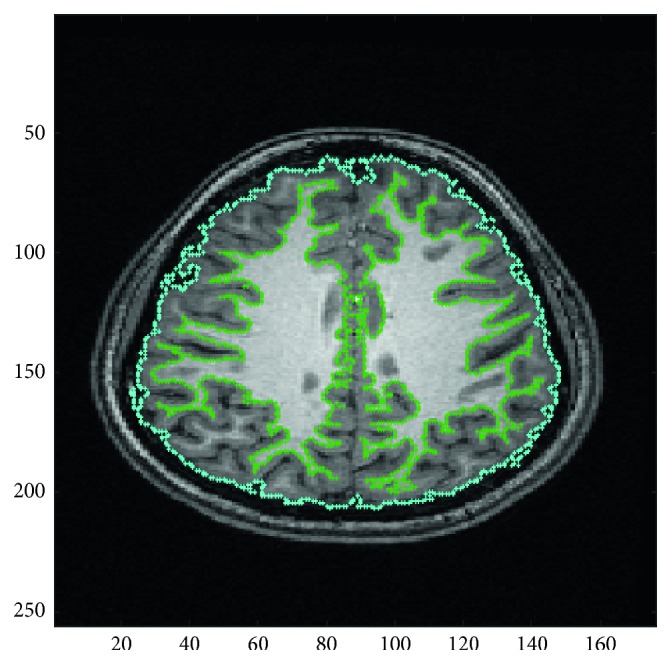
Segmented white matter (green boundary) and gray matter (blue boundary). Gray matter consists of the cortex, and its size can be measured after segmentation of the gray matter.

Segmentation of MRI images is used in many biomedical applications to effectively measure and visualize the patient's brain anatomical structures [51]. An important aspect in analyzing the brain MRI image is extracting gray and white matter regions, tumors, or lesions, which is possible through the segmentation process.  Figure 1 below shows the segmented white matter (green boundary) and gray matter (blue boundary). After segmentation of a diseased patient's MRI image, the data extracted from the multidimensional image give the information about the tumor size, type (benign or malignant), and position. This can facilitate and assist neurologists in treatment planning [52].

Initially, manual segmentation techniques were used by neurologists which are time consuming and vulnerable to human errors. Therefore, several techniques were introduced for segmentation of MRI images into regions of interest. They are classified as threshold-based, region-based, pixel classification-based, and model-based techniques. These fully automatic segmentation methods are determined by the computer without any human intervention.

In this research work, automatic segmentation of regions in cerebrum is performed using adapted fuzzy *c*-means algorithm (FCM), which is one among various pixel classification techniques, combined with connected component analysis. FCM is one amongst many predominantly used techniques for tumor segmentation and other regions in especially brain MRI scans as it gives efficient results while analyzing nonhomogeneous tumored brain MRI images [53]. This is a unique method that can also be used for noisy image segmentation to produce efficient results. Fuzzy *c*-means clustering is grouping similar data objects or components within the same cluster and dissimilar data objects within other clusters. In biomedical image processing, the term data object is nothing but pixels of an image. The same concept is implemented in this research to build a structured framework to automatically segment these cerebral regions in multidimensional brain MRI images.

Segmentation of various brain tissues is an important aspect to analyze brain image data, study patient's anatomical structure, and assist neurologists in treatment planning. Segmentation has various real-time applications such as data compression and visualization that helps neurologists to provide patient's information for surgical planning. This process of brain segmentation identifies regions of interest such as tumors, lesions, and other abnormalities. It can also be used to measure the increase or decrease in volume of tissue to measure growth of a tumor [6]. Magnetic resonance imaging (MRI) and computed tomography (CT) technologies to generate scans of internal brain structures have been increasingly used nowadays to detect tumor or any other abnormalities in human brain. These technologies make it almost compulsory for any neurologist or radiological experts to use computers in the field of medical sciences. The major goal of brain MRI segmentation is to separate the brain image into a set of important, meaningful, similar, and nonoverlapping regions having identical properties such as texture, color, intensity, or depth. The result obtained is the segmentation of each homogenous regions which are identified by labels also describing the region boundaries [7]. A typical MRI image study of one patient may require 100 or more images to be analyzed. This would be a tedious task for neurologists who have knowledge in the field to perform manual segmentation for each of the 100 images.

Nowadays, MRI imaging is used in many medical applications, especially for brain imaging to obtain clinical information and analyze patient's data. It is because, MR imaging is efficient and produces accurate results while detecting brain abnormalities of patient's brain during initial stages of any disease when compared to a CT scan. This increase in the use of MR imaging led to introducing many unsupervised automated segmentation techniques that enable managing and analyzing huge data of a patient which are in the form of an image.

Based on the repetition time (TR) and time to echo (TE), MRI scans are classified into two different sequences for scans. These scans are named as T2-weighted and T1-wieghted scans. These scans are generated depending on the time of echo (TE) and repetition time (TR) values. T2-weighted images are obtained by longer TE and TR times whereas T1-weighted images are obtained by shorter TE and TR times. The brightness and contrast of these scans are determined by T1 and T2 properties of brain tissue accordingly. The human brain contains tissues with large amounts of fat content that appear bright in MRI images. The parts of the brain which are filled with fluid appear dark in the MRI image. In our research, T1-weighted images are used because of high resolution and clarity [54].

Since the last decade, many researchers have developed advanced technologies in the field of brain MRI segmentation to detect tumors or segment brain MRI images. Even though many algorithms exist, they are not available as software packages or downloadable software and thus inaccessible to medical researchers, neurologists, surgeons, or doctors in the hospital. Even those implemented in software packages are expensive and only affordable to high-end hospitals, or do not offer the feature of automatic segmentation [55–71], or are not easy to use. However, in this, we present and publish a free-to-use graphical computational software tool that automatically performs the brain MRI image segmentation as a stand-alone application with a user-friendly easy-to-use graphical user interface and functions as a neurological disease prediction framework and disease detection tool. It is freely available to any medical student, academician, researcher, technician, nurse, doctor, neurologist, or surgeon in any country in any part of the world who accesses this paper. It is packaged in a stand-alone independent GUI, which can load medical images in any format (NIfTI, DICOM, PNG, TIF, JPG, etc.) and help neurologists to perform various automatic segmentations to analyze the patient's data. Specifically, the thickness of the cortex plays an important role in determining the severity level of dementia or cognitive impairment.

The work herein presents a method using the gray-to-white matter thickness ratio computed from the brain MRI slices of the patient as part of the development of a software platform-based computational tool for aiding neurologists in assessing anatomical and functional changes in cerebral structure from brain MRI scans of neurological patients. This GUI also enables user to perform various other actions like segmentation of brain MRI images as masks, segmented regions, or boundaries.

### 2.1. Aims and Objectives

The aims and objectives of this research paper are listed below:

1. To develop an automatic brain segmentation tool that can be used by neurologists for analyzing patient's brain image data
2. To predict neurological disease using automated segmentation to extract clinical information from the images
3. To compare automatic segmentation and manual tracings performed by experts for validation purpose

### 2.2. Step-by-Step Procedure

The stepwise procedure of this research paper is defined as follows:

1. Perform fully automatic segmentation of gray and white matter regions in brain images for disease prediction
2. Build a graphical computational tool for assisting neurologists
3. Validation of automatic segmentation with manual tracings by experts

## 3. Pixel Classification Techniques

### 3.1. Clustering Algorithms

Clustering is the grouping of objects into different clusters. In other words, the set of data is divided into subsets. Each subset should have some common property like distance, size, etc. According to the similarity measures of these data subsets, they are assigned to similar clusters. There are various clustering techniques such as fuzzy *c* clustering, each of which has their own benefits.

#### 3.1.1. *K*-Means Algorithm

The *k*-means method is one of the most widely used clustering-based algorithm for image processing. In this algorithm, an image dataset is considered which is divided into subsets or group of data. Each group of data is called cluster which is partitioned accordingly. Each cluster will have data members and cluster centroid. A point in the cluster is defined as a centroid if it has minimized sum of distances from all the data members to that point. This *k*-means is a repetitive and iterative algorithm because of which can minimize the sum of distances from all the data members to centroid and over all other clusters of the dataset. Let us assume an image data that has *a* ∗ *b* resolution and *k* be the number of clusters of that image data. Also, the pixels of the image be *P*(*a*, *b*) and c be the center point of the cluster [70, 71]. The *k*-means algorithm can be determined as follows:

After initializing the number of clusters and centroid of each cluster, compute the Euclidean distance with below formula:

(1) $$\begin{array}{c} \text{Euclidean distance}=\left|P\left(a,b\right)-C\left(k\right)\right|. \end{array}$$

In equation (1), *P*(*a*, *b*) is the input pixel at data member point (*a*, *b*) of the input image, and *C*(*k*) as in equation (2) is center for *k*th cluster.

After the calculation of distance from each pixel, determine the nearest center to all the pixels and assign the pixels to the center based on the calculated distance. Next step after assigning the pixel is to calculate again the center position of the *k*th cluster using the following formula:

(2) $$\begin{array}{c} C\left(k\right)=\frac{1}{K}\sum P\left(a,b\right). \end{array}$$

This process of computing position of centroid is repeated iteratively until error value or tolerance value is satisfied. *K*-means clustering is easy to implement and simple to understand but it also has some backlogs because of poor quality of final segmentation as the centroid value here depends on the initial value selected. This algorithm may sometimes fail as the initial value is based on the human assumptions. Therefore, many other algorithms are introduced to overcome these drawbacks.

#### 3.1.2. Fuzzy *c*-Means Algorithm

Fuzzy *c*-means clustering algorithm is the one among the most widely used methods in which the dataset is classified into clusters having similar data objects. That is, each cluster will have similar type of pixels [72]. This classification into clusters is based on the intensity values of pixels. Therefore, similar pixels are grouped into similar clusters. In this algorithm, each pixel may belong to one or more clusters unlike in *k*-means algorithm. Each pixel in the image dataset will have membership value that determines the degree of share of that pixel or data point on every cluster of that image. From this, we can build a membership matrix that has all the membership values of all the pixels of all the clusters of that image. Also, we can define the fuzzy *c*-means algorithm in other words as it processes segmentation using unique pixel classification technique in assumption that each pixel may be allowed to be present in one or more classes with value of membership that is between 0 and 1. Assume a dataset of *s* number where *X* = {*x*_1_, *x*_2_, … , *x*_*n*_}. This algorithm divides the dataset into group of fuzzy clusters according to some criteria or some condition. This grouping of data into clusters is an iterative and continuous process till all the pixels are given at least one membership of clusters based on some objective function. Given below is the objective function of fuzzy *c*-means clustering algorithm:

(3) $$\begin{array}{c} J_{m}=\overset{N}{\underset{i=1}{\sum }}\overset{c}{\underset{j=1}{\sum }}u_{ij}^{m}{\left\|x_{i}-c_{j}\right\|}^{2}. \end{array}$$

In equation (3), *m* here is a fuzzy parameter which defines the fuzziness of the clusters and *u*_*ij*_ as in equation (5) is the membership degree of cluster *C*_*j*_ which is the center of the cluster as in equation (4). The first step of the algorithm for fuzzy *c*-means clustering is to specify the number of clusters of the dataset and the matrix for the membership function of all data members of the dataset [73]. The next step is to compute the center of each cluster using the formula below:

(4) $$\begin{array}{c} C_{j}=\frac{\sum_{j=1}^{n}u_{ij}^{m}x_{i}}{\sum_{j=1}^{n}u_{ij}^{m}}. \end{array}$$

After the center calculation, one should determine the error or cost value and evaluate if it is less than the threshold value so that to improve the previous iteration of the function. If the error value is satisfactory then it is further processed to cluster the data. If the error value is not satisfactory, membership matrix is continuously updated till the results are satisfactory to obtain final segmentation with improved level of quality. Below is the condition to compute the relation with membership function:

(5) $$\begin{array}{c} u_{ij}=\frac{1}{\sum_{k=1}^{c}{\left[d_{ij}/d_{kj}\right]}^{\left(2/\left(m-1\right)\right)}}. \end{array}$$

There are many other segmentation algorithms among which this fuzzy *c*-means algorithm is more suitable to analyze patient's data through segmentation process. In this research work, we use an adaptive fuzzy *c*-means clustering algorithm for segmentation of gray and white matter regions in brain MRI images.

## 4. Brain MRI Segmentation

Past literature presents reduction (measured as atrophy rate) of cortex volume as a valid measure for dementia from patient MRI scans. The estimation of atrophy rate requires measurement of the gray and white matter regions in the brain MRI images of the patient. In the proposed method, the gray and white matter are automatically segmented using a form of adaptive modified pixel clustering methods such as *k*-means or fuzzy *c*-means clustering, which will cluster the pixels by labeling them (based on their intensities) to belong to the gray matter, white matter, cerebrospinal fluid, or background [74]. The adaptive clustering methods are modified by running them separately for the gray and white matter and postprocessing with connected region labeling to separately label the gray and white matter regions.

### 4.1. Image Acquisition

The patient's brain MRI image and neurological data used in this research work were obtained from the Image and Data Archive (IDA) powered by Laboratory of Neuro Imaging (LONI) provided by the University of Southern California (USC) and also from the Department of Neurosurgery at the All India Institute of Medical Sciences (AIIMS), New Delhi, India. The data were anonymized as well as followed all the ethical guidelines of the participating research institutions.

### 4.2. Segmentation Methodology

**Figure 2:**
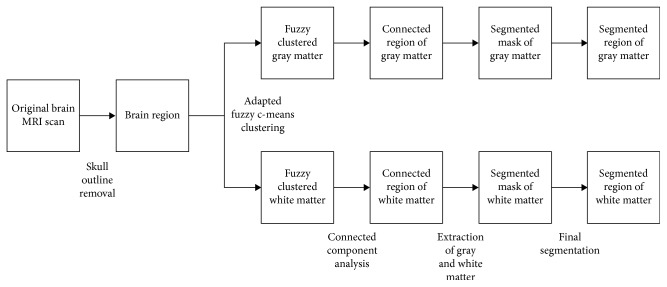
Block diagram of this paper's proposed fully automatic brain MRI gray and white matter segmentation procedure.

**Figure 3:**
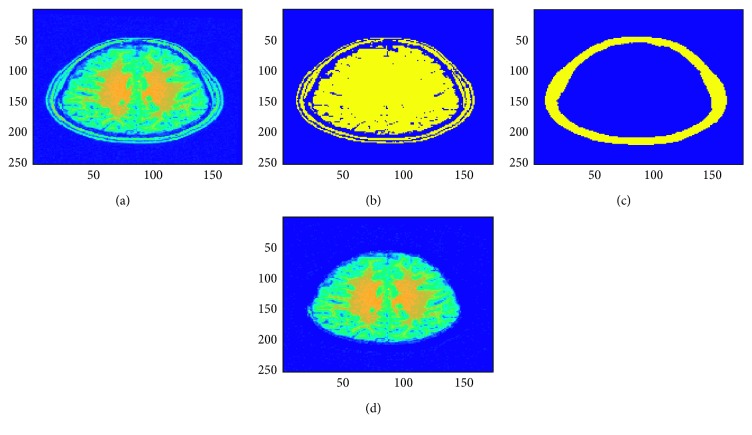
Skull outline detection in brain MRI images: (a) original MRI image slice; (b) thresholded MRI image slice; (c) detected skull outline; (d) skull outline removed.

The methodology for segmenting the gray and white matter used in this research is illustrated in Figure 2. The first step is the removal of the skull outline from the brain MRI images with the Hough transform. Fuzzy *c*-means clustering is next applied on the skull outline removed brain MRI image slice to obtain separate clustered image slices for the gray and white matter regions. These clustered gray and white matter images are divided into connected regions using connected component labeling. The largest two connected regions are heuristically the gray and white matter regions. The binary extracted gray and white matter images can be used as masks, which when applied to the original brain MRI image produces the final segmented gray and white matter regions with the original pixel intensities [75]. The skull outline removal using the Hough transform is shown in Figure 3. The detected skull outline is removed to obtain only the cerebral cortex in the MRI image slice. This cerebral cortex image slice is used in the fuzzy *c*-means clustering step of the procedure.

In this paper, we present a framework for neurological disease prediction and decision making for patients of cognitive impairment, dementia, or Alzheimer's disease based on automatic segmentation of gray and white matter regions as anatomical features in brain MRI images. Changes in the size or volume of these regions can be correlated to changes in cerebral structure in patients with Alzheimer's, dementia, cognitive impairment, or other neurological disorders. Specifically, the thickness of the cortex plays an important role in determining the severity level of dementia or cognitive impairment [76]. The work herein presents a method using the segmentation of gray and white matter from the brain MRI slices of the patient as part of the development of a software platform-based computational tool for aiding neurologists in assessing anatomical and functional changes in cerebral structure from brain MRI scans of neurological patients. The aforementioned tool can be implemented as a software package that can be installed in the computational platforms in the neurology department or division of hospitals. In its final implementation and deployment, this tool would predict neurological disease type and severity after automatically processing the brain MRI or CT images with the abovementioned algorithms and displaying the highlighted gray and white matter regions in the brain CT or MRI images [77].

In the field of medical image processing, the most challenging task to any neurologist or a doctor or a scientist is to detect the patient's disease by analyzing the patient's clinical information. Patient's data is extracted and analyzed to detect the abnormalities and to measure the illness of the disease which helps a medical practitioner to cure the disease at its early stages [78]. Extraction of brain abnormalities in brain MRI images is performed by segmentation of gray and white matter regions in patient's brain MRI images. After segmentation is performed, patient's clinical data such as the area of the cortex, size of tumor, type of tumor (malignant or benign) and position of tumor are determined which helps a doctor to take early decisions for surgery or treatment to cure any brain disease.

During initial days, these segmentation techniques were performed manually by subject matter experts or neurological experts which consumes time and effort of neurological specialists in the field. The segmentation results obtained from the manual segmentation techniques may not be accurate due to vulnerable and unsatisfactory human errors which may lead to inappropriate surgical planning. Therefore, it has become very much necessary for a neurologist or an academician or a researcher to introduce automatic segmentation [79, 80] techniques, which give accurate segmentation results. These segmentation techniques that are performed automatically are of two types typically known as semiautomatic and fully automatic segmentation techniques. In a semiautomatic segmentation process, partial segmentation is performed automatically and then the results thus obtained are checked by neurological experts to modify for obtaining final segmentation results. In a fully automatic segmentation technique, there is no need for manual checking by neurological experts which minimizes his time and effort. These fully automatic segmentation techniques are classified as threshold-based, region-based, pixel classification-based, and model-based techniques which are determined by the computer without any human participation.

This research work presents the segmentation of various regions that are segmented automatically using a technique called fuzzy *c*-means algorithm (FCM), which is a pixel classification technique followed by component labeling technique which is used widely in biomedical image processing to perform fully automatic segmentation in brain MRI images [81].

Over the past few years, a set of techniques were introduced for automatic image segmentation among which fuzzy *c*-means (FCM) clustering method yields both gray matter and white matter regions more homogenously which can efficiently remove noisy spots when compared to other segmentation techniques. Figure 2 shows the detailed description of the segmentation process as a block diagram.

Therefore, this technique can be used to segment noisy brain MRI images obtaining accurate, reliable, and robust results. Also, unlike other techniques, this can be used for both single-featured and multifeatured information analysis with spatial data. This automated unsupervised technique can be used to perform segmentation to achieve feature analysis, clustering, and classifier designs in fields of astronomy, target recognition, geology, medical imaging, and image segmentation [9]. A set of data points constitutes to form an image that has similar or dissimilar regions. This algorithm helps to classify the similar data points into similar clusters by grouping them based on some similarity criteria. In medical image processing field, image pixels are highly correlated as they may have same characteristics or feature data to its next or immediate neighbor. In this method, spatial information of neighboring pixels is highly considered while performing clustering. This paper presents a technique for clustering of brain MRI image slices into different classes followed by component labeling using knowledge-based algorithm. The steps in the fully automatic segmentation algorithm are as follows.

### 4.3. Skull Outline Detection

The preliminary step in our research is to extract the skull outline from an MRI image slice as it is not our region of interest. Also, these quantitative studies especially in living organisms of brain MRI images usually will have a preparatory processing in which the part of the brain itself is isolated from the external brain regions and no-brain tissues which are not required for brain analysis. This process of skull outline detection and removal is called skull stripping. This helps us to focus more on the actual brain itself [10]. In this stage, many superfluous and nonbrain tissues such as fat, skin, and skull in brain images had been detected and removed using Hough Transform which is an image feature extraction tool in digital image processing. This Hough transform technique for skull outline detection helps to find unwanted points or data objects of an image with different shapes such as circular and elliptical using voting procedure in a parameter space. These generalized Hough transform techniques are used to detect an arbitrary shape at a given position and scale. In this technique, in a parametric space of an MRI image, parametric shapes are detected by tracing the acquisition of various points in the space. If in an image a shape like circle and elliptical exists, all its points are mapped in the parametric space grouping them together around the parametric values forming clusters which correspond to that shape [11]. The result obtained in this step is shown in Figure 3.

### 4.4. Adaptive Fuzzy *c*-Means Clustering

After the skull outline detection and removal, internal part of the brain is clustered into different regions. Clustering is a well-known and widely used technique for pattern classification and image segmentation purposes in the field of medical sciences. In this process, similar data objects or pixels are grouped into similar clusters. Usually, medical images tend to have more noise due to its internal and external factors. During the segmentation process, the medical images having noise generate inefficient results, and it is difficult to analyze anatomical structures of patient's brain [12]. This may lead to inappropriate diagnosis and treatment planning. Therefore, to avoid inaccurate results during segmentation process, several types of image segmentation techniques were introduced by the researchers and neurologists to achieve accurate results during segmentation of regions in an MRI image of a patient. These techniques can perform segmentations equally for noise MRI images [13–18]. Among them, fuzzy *c*-means clustering methods are widely used techniques in MRI segmentation as they have substantial advantages comparatively because of uncertainty present in brain MRI image data. To enhance features of fuzzy *c*-means algorithm, in our research, adaptive fuzzy *c*-means clustering algorithm is used as it minimizes computational errors [19].

### 4.5. Connected Component Labeling

In the next step, the clustered image is subjected to connected component labeling based on connectivity. Deriving and labeling positions of several disjoint and connected components in brain MRI image is a very essential step in segmentation process [20]. In any medical image, pixels which are positioned together as connected components will have similar values for their intensities. Connected component labeling method scans the image, pixel-by-pixel to first detect the connected component pixels and then it extracts connected pixel regions which are adjacent to one another. These pixels which positioned together will have same set of intensity values [21–25]. After all groups have been extracted, each pixel component is labeled according to component it was assigned to. In our research, we use 8-connectivity measures for connected component labeling.

### 4.6. Final Segmentation Mask after Removing Noise

**Figure 4:**
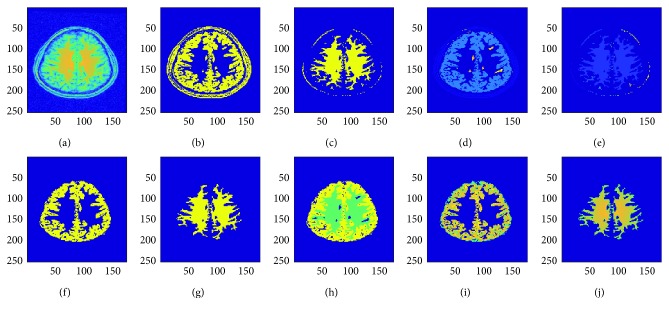
Fully automatic gray and white matter segmentation in brain MRI images (for a sample patient specimen image). (a) Original MRI frame. (b) Fuzzy gray matter. (c) Fuzzy white matter. (d) Connected gray matter. (e) Connected white matter. (f) Segmented gray matter. (g) Segmented white matter. (h) Gray and white matter. (i) Gray matter mask. (j) White matter mask.

**Figure 5:**
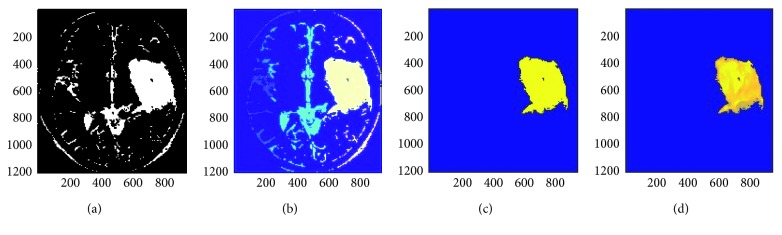
Tumor in brain region segmentation in a sample tumor brain MRI image. The brain MRI image after performing fuzzy *c*-means and connected regions operations is shown along with the final segmented tumor region and mask using the fully automatic procedure for tumor segmentation from the brain segmentation. This shows that the method proposed in this paper successfully works for tumor segmentation and identification along with gray and white matter segmentation. Thus, brain tumor segmentation is another application of this paper's proposed algorithm along with gray and white matter region segmentation. (a) Fuzzy tumor region. (b) Connected tumor region. (c) Segmented tumor region. (d) Tumor region mask.

The final step is to obtain actual segmented gray and white matter regions by overlaying gray matter and white matter masks on original MRI image to remove all pixels which background and only keep the pixels in the foreground or regions of interest in the original image [26]. This method enhances the distinction of gray and white matter regions and allows more accurate segmentation results. The algorithm presented herein works for gray and white matter segmentation as well as tumor segmentation in brain MRI images.  Figure 4 below shows the results on a sample patient specimen brain MRI image obtained from the abovementioned fuzzy *c*-means clustering followed by the connected component labeling to extract the cerebral regions as masks [27, 28]. When these masks are applied to the original image, final gray and white matter regions segmentation or tumor segmentation results are obtained. The results thus obtained are shown in Figure 4 below for a normal patient brain MRI image. As this method is also applicable for tumor segmentation, Figure 5 shows the results of tumor segmentation applying this work's proposed algorithm on a tumor brain MRI image.

The segmentation results for a brain tumor patient's brain MRI images are shown below. The figures below show a sample brain MRI image of a patient brain with a tumor. These figures demonstrate that the algorithm developed herein for detection of gray and white matter regions works well for tumor detection and segmentation of the tumor section in a patient's brain as well. As mentioned earlier, in our segmentation methodology after skull outline detection, we perform adapted fuzzy *c*-means clustering followed by the connected component labeling to extract the gray and white matter regions as masks for gray and white matter segmentation or to extract the brain region and tumor regions as masks for tumor segmentation and identification.

**Figure 6:**
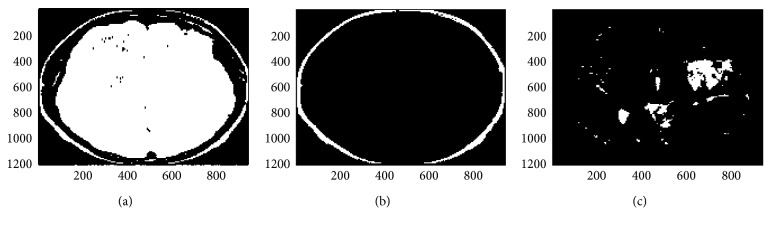
Skull outline detection in brain MRI image with tumor. (a) Threshold MRI image Slice. (b) Detected skull outline. (c) Skull outline removed.

The results of the automatic segmentation algorithm for tumor identification and segmentation on a sample patient's tumor brain MRI image are shown below in this section. The first step was skull outline removal (see Figure 6), and the final segmentation results of this brain tumor MRI image are shown in Figure 5.

Table 1 shows the comparison of different brain MRI segmentation methods [81, 82] based upon pixel classification and clustering classified by the region of interest being segmented.

## 5. Segmentation Tool

To process, extract and analyze the patient's image data, a neurologist or a researcher requires a computational tool that can perform all the required functions automatically minimizing the cost, effort, and time. These software tools are widely used nowadays in almost all the hospitals to detect patient's disease by analyzing patient-specific information and to provide patient-specific medical care at early stages of the disease [29]. These days, software engineers and programmers have been actively developing tools which are used in medical fields to assist neurologists, scientists, doctors, and academicians to analyze patient specific information. This research work herein presents an independent standalone graphical computational tool which is developed for assisting neurologists or researchers in the field to perform automatic segmentation of gray and white matter regions in brain MRI images [30, 31]. This software application is built using a neurological disease prediction framework for diagnosis of neurological disorders like dementia, impairment, brain injury, lesions, or tumors in patient's brain. This tool provides the user to perform automatic segmentation and extract the gray and white matter regions of patient's brain image data using an algorithm called adapted fuzzy *c*-means (FCM) [32]. In this research work, we also present the methodology used to obtain segmentation, in which patient's images are subjected to fuzzy *c*-means clustering followed by connected component labeling technique.

The entire process of feature extraction, classification, preprocessing, and segmentation [33] is developed as a graphical computational tool with a user interface (GUI). This application built is a stand-alone graphical user interface (GUI) that will load the brain MRI images from the local computers of neurologists on the click of a button and then segment out [34–37] the gray and white matter regions in the brain MRI images upon just the click of buttons and display the results as a mask, color images, or as the boundaries of those two cerebral regions. The developed GUI system assists neurologists or any user making it easy to upload patient's brain image from his local computer, viewing and obtaining the results in very less time, reducing efforts due to manual tracings by the experts [38–42]. The GUI has the following features:

1. Automatized segmentation of brain MRI images is provided as a stand-alone independent software package.
2. It is freely accessible to all researchers in the medical field and neurologists, radiologists, and doctors in any part of the world.
3. It is user-friendly and easy to use.
4. It automatically segments the brain images and so no manual tracing is required by the user. This tool allows timely efficient segmentation of the brain MRI images so that the neurologists' or neurosurgeons' precious time is used efficiently and not wasted on manual segmentation.
5. It is developed to support several medical image datatypes (NIfTI, DICOM, PNG, etc.).
6. Neurological disease prediction framework can be provided in this software tool.
7. The tool was developed in collaboration with neurosurgeons and neurologists at the All India Institute of Medical Sciences (AIIMS) and hence it has the expert neurological feedback and opinion of doctors implemented in it.

**Figure 7:**
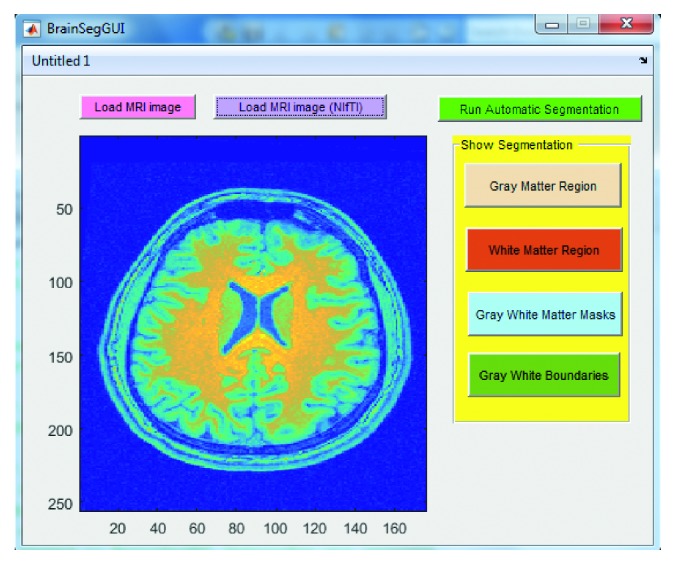
Screenshot of the graphical user interface (GUI) designed and developed in this work for automatic brain MRI image processing. Step shown here is to load the MRI image (NIfTI in this case) upon the click of the “Load MRI image” or “Load MRI image (NIfTI)” button depending upon the image type.

**Figure 8:**
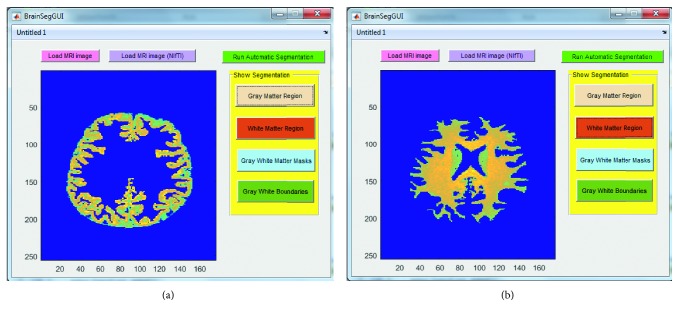
Screenshots of the graphical user interface (GUI) designed and developed in this work for automatic brain MRI image processing. Steps shown here are to show extracted gray (a) and white (b) matter regions upon the click of the “Gray Matter Region” (a) and “White Matter Region” (b) buttons, respectively.

**Figure 9:**
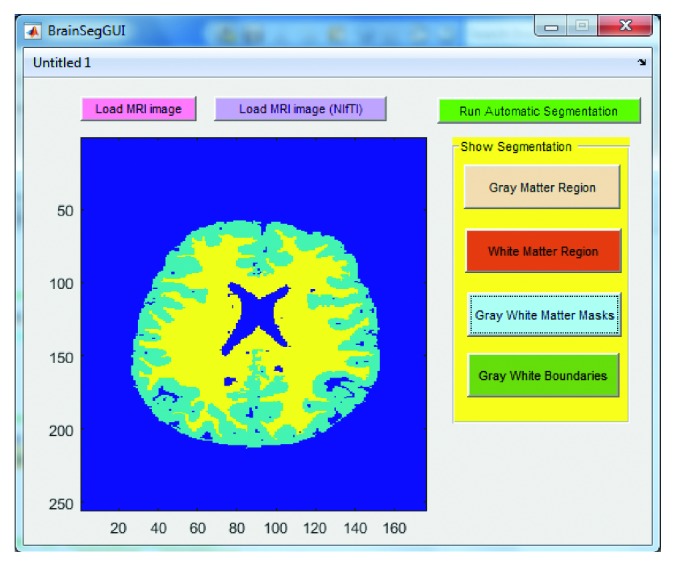
Screenshot of the graphical user interface (GUI) designed and developed in this work for automatic brain MRI image processing. Step shown here is to show the gray and white matter masks upon the click of the “Gray White Matter Masks” button.

**Figure 10:**
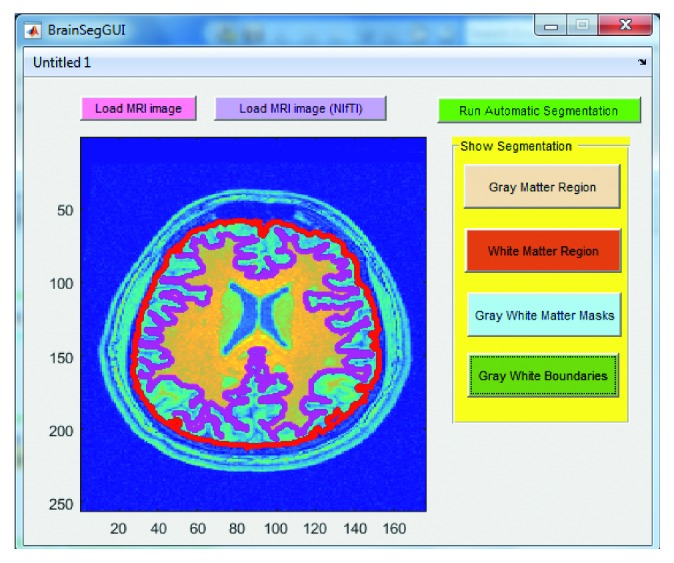
Screenshot of the graphical user interface (GUI) designed and developed in this work for automatic brain MRI image processing. Step shown here is to show the gray matter boundary (shown as a red colored contour) and white matter boundary (shown as a magenta colored contour) superimposed on the original brain MRI image upon the click of the “Gray White Boundaries” button.

Below are the three screenshots which show running the GUI for loading the brain MRI image (Figure 7), viewing the gray and white matter segmented regions (Figure 8), viewing the gray and white matter extracted masks (Figure 9), and viewing the gray and white matter region boundaries (Figure 10).

## 6. Manual Segmentation

**Figure 11:**
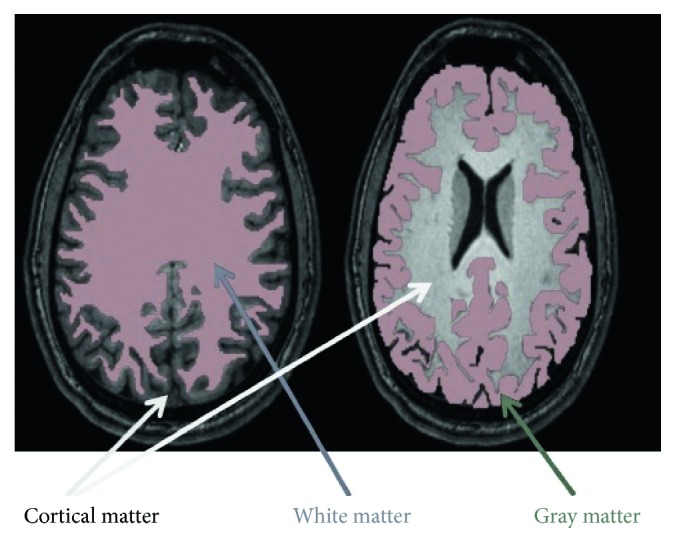
Sample manual segmentation (labeling) by neurologist expert of the gray and white matter regions in brain MRI images: white matter region (left) and gray matter region (right).

In this section, the accuracy of the proposed automatic segmentation methodology of the white and gray matter regions was validated against manual neurological tracing-based segmentation by experts. The validation of the automatic segmentation of gray and white matter regions in patient brain MRI images using adapted fuzzy *c*-means clustering followed by the connected labeling is done by verifying against the manual segmentation by neurologist experts shown in Figure 11.

**Figure 12:**
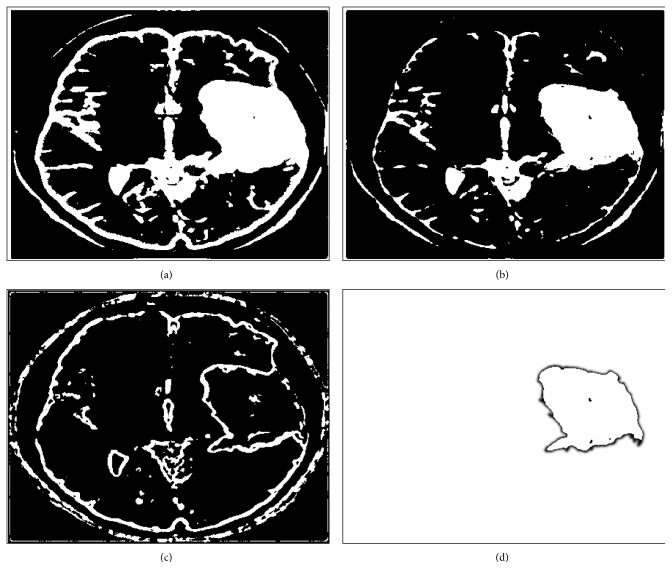
Example of steps in segmentation (tracing) by expert of the gray and white matter regions in brain tumor MRI images in a sample patient brain MRI image.

We have also performed validation of the automatic segmentation of gray and white matter and tumors in tumor brain MRI images using adapted fuzzy *c*-means clustering combined with the connected component labeling and this is validated by the manual segmentation by experts, an example of which is shown in Figure 12.

## 7. Validation

**Figure 13:**
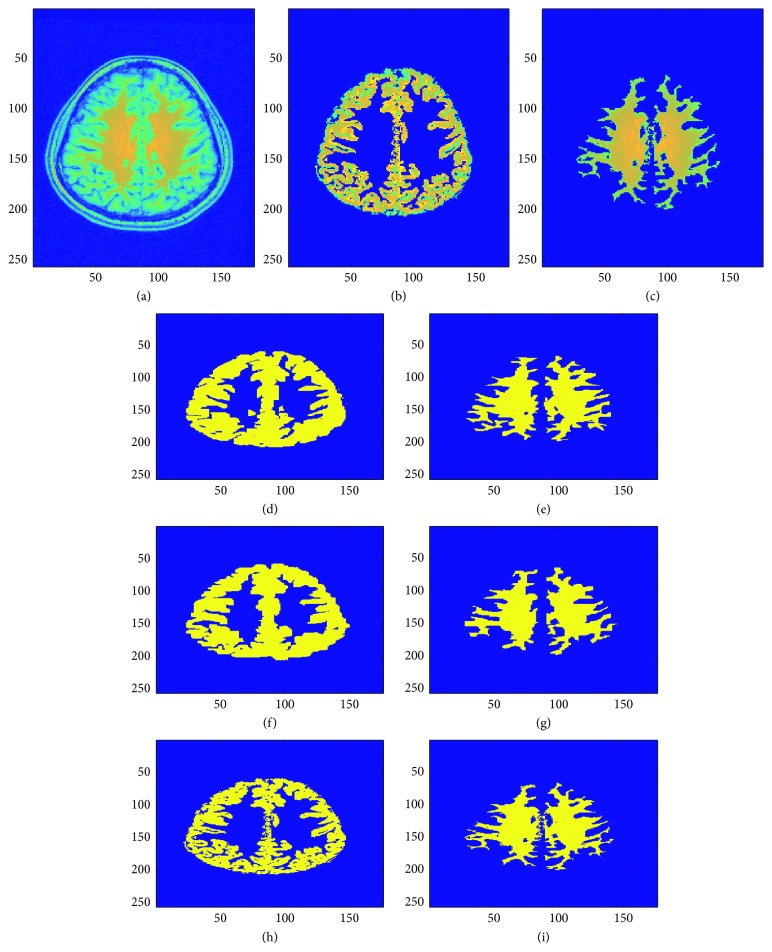
Visual comparison of two manual expert tracing-based and automatic segmentation (using the fully automatic segmentation method presented in this paper) results of sample patient 1 brain MRI image (see last row of Table 2 and Figure 16 for validation results that show the high accuracy and low error of the automatic segmentation method proposed in this research as compared to the two manual expert tracing-based segmentation results). (a) Original brain MRI image. (b) Gray matter region in original image. (c) White matter region in original image. (d) Gray matter: manual segmentation 1. (e) White matter: manual segmentation 1. (f) Gray matter: manual segmentation 2. (g) White matter: manual segmentation 2. (h) Gray matter region: automatic segmentation. (i) White matter region: automatic segmentation.

**Figure 14:**
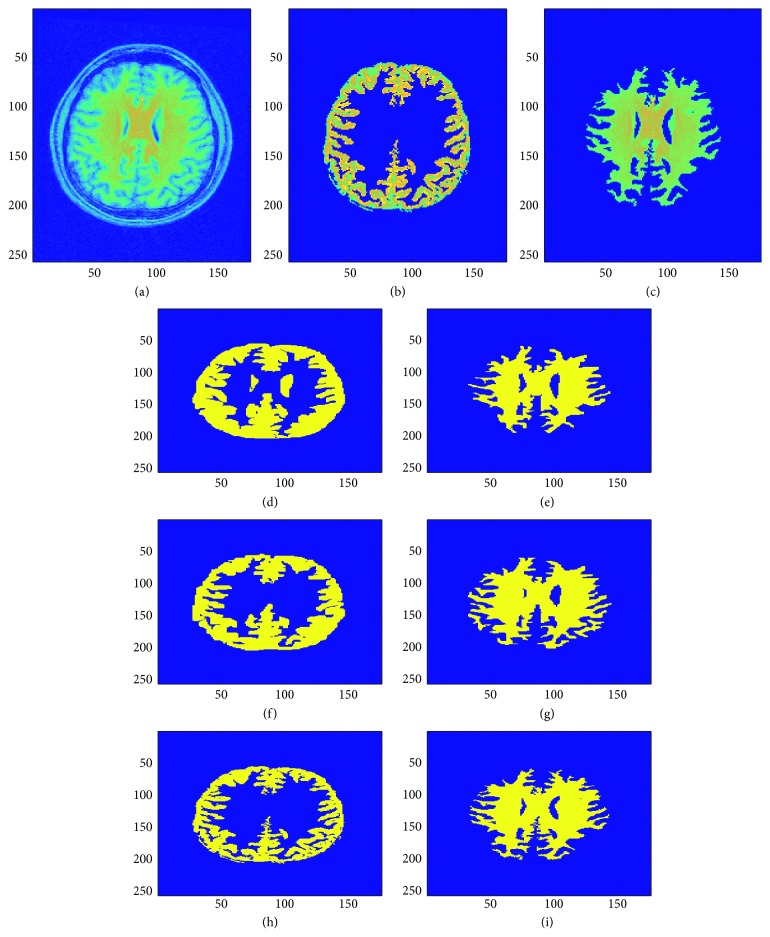
Visual comparison of two manual expert tracing-based and automatic segmentation (using the fully automatic segmentation method presented in this paper) results of sample patient 2 brain MRI image (note the difference between the two manual segmentations of the gray matter: one including and the other excluding portion(s) of the cerebrospinal fluid region; this shows the robustness of the proposed automatic segmentation algorithm to still have high validity even when considering error, taking human manual error into account; see last row of Table 2 and Figure 16 for validation results that show the high accuracy and low error of the automatic segmentation method proposed in this research as compared to the two manual expert tracing-based segmentation results). (a) Original brain MRI image. (b) Gray matter region in original image. (c) White matter region in original image. (d) Gray matter: manual segmentation 1. (e) White matter: manual segmentation 1. (f) Gray matter: manual segmentation 2. (g) White matter: manual segmentation 2. (h) Gray matter region: automatic segmentation. (i) White matter region: automatic segmentation.

**Figure 15:**
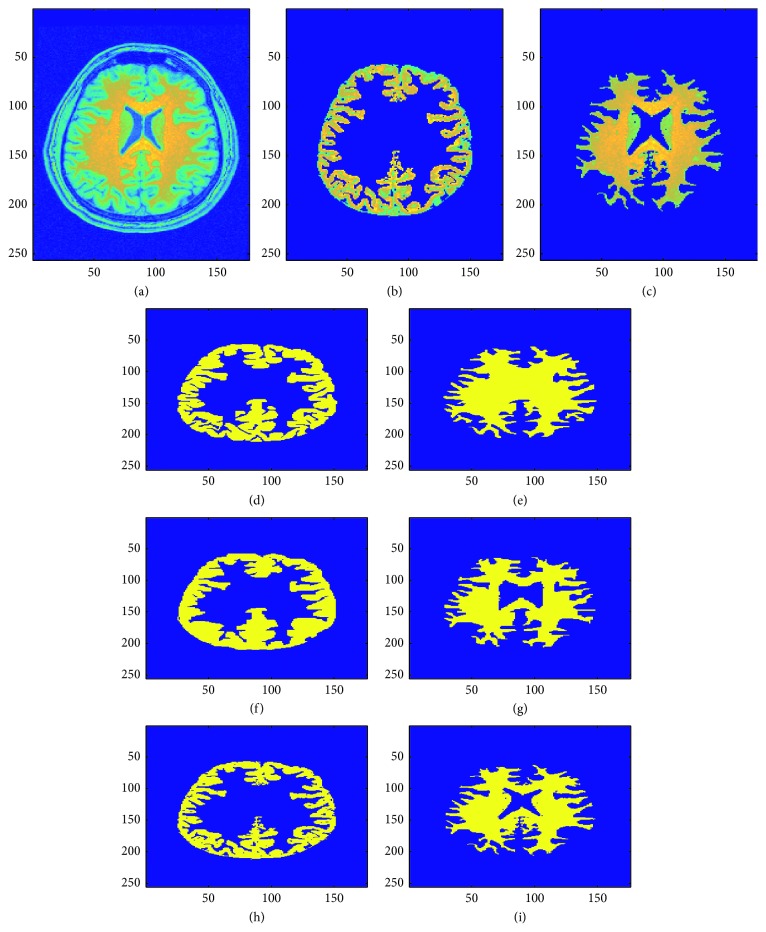
Visual comparison of two manual expert tracing-based and automatic segmentation (using the fully automatic segmentation method presented in this paper) results of sample patient 3 brain MRI image (see last row of Table 2 and Figure 16 for validation results that show the high accuracy and low error of the automatic segmentation method proposed in this research as compared to the two manual expert tracing-based segmentation results). (a) Original brain MRI image. (b) Gray matter region in original image. (c) White matter region in original image. (d) Gray matter: manual segmentation 1. (e) White matter: manual segmentation 1. (f) Gray matter: manual segmentation 2. (g) White matter: manual segmentation 2. (h) Gray matter region: automatic segmentation. (i) White matter region: automatic segmentation.

**Figure 16:**
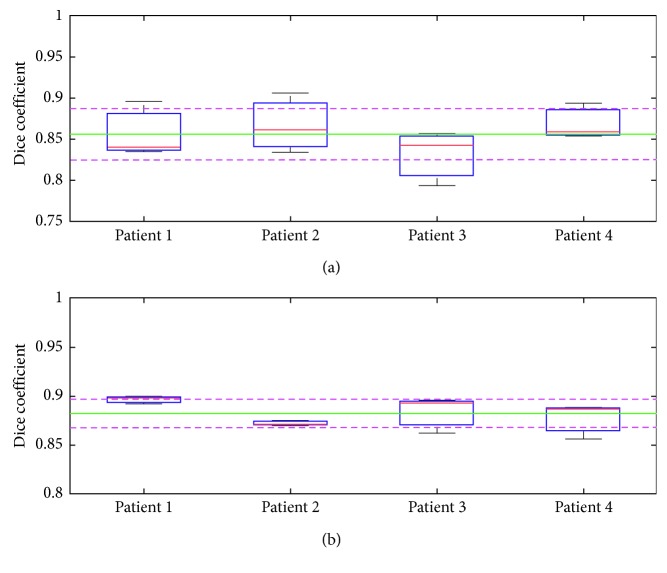
Box plots for Dice coefficients to compare manual and automatic segmentation of brain MRI images of 5 patients. Overall mean of the Dice coefficient is represented as a green line and standard deviation is represented as the dashed purple lines. (a) Comparison between automatic and manual segmentations of gray matter. (b) Comparison between automatic and manual segmentations of white matter.

This validation compares the manual and automatic segmentation of five patient brain MRI images statistically using the Dice coefficient as a similarity measure [79, 80, 84–87]. Figures 13, 14, and 15 show the sample manual and automatic segmentation of three of the patients. For this purpose, a total of five MRI scans of different patients were used to validate the automatic segmentation proposed in this paper by comparison against manual segmentation by neurological experts for each patient's MRI image by calculating the [89–95] Dice coefficient between the automatic and manual segmentation for each of the patient brain MRI images. For each patient brain MRI image, manual segmentation was performed three times by experts. The Dice coefficients are calculated between all the manual and automatic segmentation for each patient brain MRI image.  Figure 16 shows the box plots of the Dice coefficients calculated as the similarity measure to compare manual and automatic segmentation of the brain MRI images for the five sample patients.

The box plots in Figure 16 show the minimum, first quartile, median, third quartile, and maximum values of the distribution of Dice coefficients computed between each pair of manual and automatic segmentation for each patient. Each patient's brain MRI image was automatically segmented by the algorithm proposed in this research work and was manually traced three separate times by experts (three manual segmentations) [96–102]. So, several Dice coefficients were calculated between each of the manual segmentations by expert tracing and the automatic segmentation for each patient.

One of the challenging tasks in medical imaging sciences is to extract the gray and white matter from MRI brain images. In our research, we have used adaptive fuzzy *c*-means algorithm in which pixels are classified based on intensity and membership-based fuzzy *c*-means clustering with preprocessing using elliptical Hough transform and postprocessing using connected region analysis.  Table 2 shows the average Dice coefficient values for the similarity measures between the manual expert tracings and the automatic segmentations of gray matter, white matter, and total cortical matter results of the proposed algorithm presented in this paper, compared with previously used standard state-of-the-art methods for brain MRI segmentation. The proposed algorithm presented in this work has the highest Dice coefficient similarity measures for gray, white, and total cortical matter segmentation when compared with other previously published standard state-of-the-art brain MRI segmentation methods.

## 8. Future Work

Future research in this work will further investigate gray white matter ratio as a marker of cognitive impairment or dementia. The advantage of this proposed future idea is that it will not require a sequence of MRI scans over several dates but will rather be able to predict severity of cognitive impairment or dementia from a single MRI scan.

The motivation of this work is that this idea is implemented in this proposed user-friendly software platform with an easy-to-use graphical user interface for neurologists to automatically quantify severity of dementia or cognitive impairment from a single structural MRI scan of a patient brain. In future, the proposed algorithm will be applied on larger datasets of brain MR images for gray and white matter extraction, which can be validated by experts. Further, neurological disease classification can be done based on volume ratio of gray and white matter for different MRI images.

The idea proposed herein is that the machine learning or model-based prediction algorithm that is developed can calculate the cognitive impairment level as the distance from the regression line, which here is the curve fitted to the scatter data points in the gray white matter ratio to age plot from previously published research.

**Figure 17:**
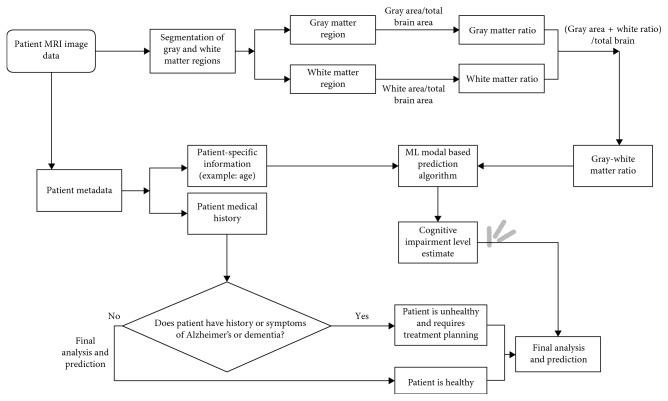
Neurological disease prediction and decision-making framework for determining cognitive impairment level based on gray and white matter ratio and patient data.

Figure 17 shows a depiction of the neurological disease prediction and decision-making framework developed in this work for prediction of cognitive impairment level. The patient image data and metadata containing the age and medical history are also employed. A model-based prediction or machine learning algorithm can be used to output the prediction based on the input parameters, namely, age and gray-white matter ratio. This algorithm can be based on previous research published on the correlation between age and gray and white matter ratios.

As proposed in this work, the average thickness and volume measurements of the neocortical and nonneocortical regions between the boundaries of the white and gray matter regions, the aggregate of the parts of the regions in both the left and right hemispheres, can be used as the measures with which the cognitive impairment or dementia is quantitatively assessed for a patient, based on their brain MRI scan.

As shown in Figure 17, based on the work proposed in this research paper, a neurological disease detection and decision-making framework can be developed with segmentations of the gray and white matter regions to determine the level of atrophy or degeneration in the cortical matter and assess the severity of dementia or cognitive impairment in a neurologically diseased patient.

## 9. Conclusion

The research presented in this work facilitates efficient and effective automatic segmentation of gray and white matter regions from brain MRI images, which has several clinical neurological applications. A fully automatic segmentation methodology using elliptical Hough transform along with pixel intensity and membership-based adapted fuzzy *c*-means clustering followed by connected component labeling and region analysis has been implemented in this research to perform segmentation of gray and white matter regions in brain MRI images. The algorithm was tested and verified for several sample brain MRI images including patient brain MRI images having tumor sections. The algorithm implemented in this research acquired higher accuracy in the results when compared to other previous state-of-the-art algorithms that have been published so far. Manual segmentations were performed by neurological experts for several patient brain MRI images. These manual segmentations were used to compare and validate with the results obtained from the automatic segmentations in this research work. Validations were performed by calculating several Dice coefficient values between the automatic segmentation results and the manual segmentation results. The Dice coefficient values are similarity measures that are represented statistically using box plots in this research. The average of the Dice coefficient values obtained was higher for the algorithm proposed and implemented in this work when compared to other methodologies that have been published so far in the medical field to automatically segment gray and white matter regions in brain MRI images. The automatized computational segmentation tool developed in this research can be employed in hospitals and neurology divisions as a computational software platform for assisting neurologist in detection of disease from brain MRI images after MRI segmentation. This tool obviates manual tracing and saves the precious time of neurologists or radiologists. This research presented herein is foundational to a neurological disease prediction and disease detection framework, which in the future, with further research work, can be developed and implemented with a machine learning model-based prediction algorithm to detect and calculate the severity level of the disease, based on the gray and white matter region segmentations and estimated gray and white matter ratios to the total cortical matter, as outlined in this research.

## Figures and Tables

**Table 1:** Comparison of different brain MRI segmentation methods [81, 82] along with method proposed by the authors [83] based upon pixel classification and clustering classified by the region of interest being segmented.

Region of interest	Method	Procedure
Brain tumors	*k*-means + fuzzy *c*-means	Pixel intensity *k*-means followed by pixel intensity and membership-based fuzzy *c*-means clustering with preprocessing using median filters and postprocessing using feature extraction and approximate reasoning
Brain lesions	Fuzzy *c*-means with edge filtering and watershed	Pixel intensity and membership-based fuzzy *c*-means with preprocessing using thresholding techniques and postprocessing using edge filtering and watershed techniques
Gray and white matter regions	Adaptive fuzzy *c*-means (proposed method in this work)	Pixel intensity and membership-based fuzzy *c*-means clustering with preprocessing using elliptical Hough transform and postprocessing using connected region analysis

**Table 2:** Performance and accuracy comparison of the authors' proposed automatic brain MRI segmentation algorithm [83] with previous algorithms [88] using Dice coefficients as similarity measure estimated between manual expert tracings and automatic algorithm-based segmentation.

Methods	Procedure	Average of Dice coefficients (gray matter)	Average of Dice coefficients (white matter)	Average of Dice coefficients (total cortical matter)
*K*-means	Statistical distance-based *k*-means clustering with preprocessing using median filters	0.70	0.71	0.71
Intensity-based fuzzy *c*-means	Pixel intensity and membership-based fuzzy *c*-means clustering with preprocessing using median filters	0.71	0.79	0.75
Adaptive fuzzy *c*-means with preprocessing and postprocessing (proposed method in this work)	Pixel intensity and membership-based fuzzy *c*-means clustering with preprocessing using elliptical Hough transform and postprocessing using connected region analysis	0.86	0.88	0.87

## Data Availability

The data can be provided to the readers from the corresponding author upon request and can also be sent to them along with the code and software to test out and see the results for themselves.
